# *N*′-[(*E*)-2,6-Dichloro­benzyl­idene]pyrazine-2-carbohydrazide

**DOI:** 10.1107/S1600536809053343

**Published:** 2009-12-16

**Authors:** R. Alan Howie, Marcus V. N. de Souza, Solange M. S. V. Wardell, James L. Wardell, Edward R. T. Tiekink

**Affiliations:** a Department of Chemistry, University of Aberdeen, Old Aberdeen, AB15 5NY, Scotland; b Fundação Oswaldo Cruz, Instituto de Tecnologia em Farmacos - FarManguinhos, Rua Sizenando Nabuco, 100, Manguinhos, 21041-250 Rio de Janeiro, RJ, Brazil; c CHEMSOL, 1 Harcourt Road, Aberdeen AB15 5NY, Scotland; d Centro de Desenvolvimento Tecnológico em Saúde (CDTS), Fundação Oswaldo Cruz (FIOCRUZ), Casa Amarela, Campus de Manguinhos, Av. Brasil 4365, 21040-900 Rio de Janeiro, RJ, Brazil; e Department of Chemistry, University of Malaya, 50603 Kuala Lumpur, Malaysia

## Abstract

The title compound, C_12_H_8_Cl_2_N_4_O, is non-planar, the dihedral angle formed between the pendant pyrazine and benzene rings being 12.55 (11)°. An intra­molecular N—H⋯N hydrogen bond occurs. The amide groups self-associate *via* N—H⋯O hydrogen bonding, forming supra­molecular chains with base vector [101], which are stabilized by C—H⋯O contacts. C—H⋯N inter­actions are formed orthogonal to the chains.

## Related literature

For background to the biological activity of pyrazine derivatives, see: Barlin (1982 ▶); Dolezal *et al.* (2002 ▶); Krinkova *et al.* (2002 ▶); Özdemir *et al.* (2009 ▶); Chaisson *et al.* (2002 ▶); Gordin *et al.* (2000 ▶); de Souza *et al.* (2005 ▶). For related structures, see: Wardell *et al.* (2008 ▶); Baddeley *et al.* (2009 ▶).
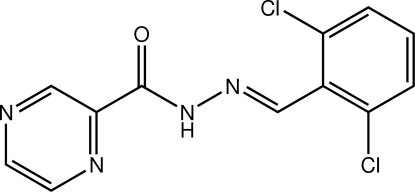


## Experimental

### 

#### Crystal data

C_12_H_8_Cl_2_N_4_O
*M* *_r_* = 295.12
Monoclinic, 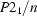

*a* = 6.9325 (3) Å
*b* = 24.5997 (13) Å
*c* = 7.6136 (4) Å
β = 111.709 (3)°
*V* = 1206.31 (10) Å^3^
*Z* = 4
Mo *K*α radiation
μ = 0.53 mm^−1^
*T* = 120 K
0.26 × 0.08 × 0.02 mm

#### Data collection

Nonius KappaCCD area-detector diffractometer
Absorption correction: multi-scan (*SADABS*; Sheldrick, 2007 ▶) *T*_min_ = 0.760, *T*_max_ = 1.000
8211 measured reflections
2108 independent reflections
1858 reflections with *I* > 2σ(*I*)
*R*_int_ = 0.052

#### Refinement

*R*[*F* ^2^ > 2σ(*F* ^2^)] = 0.052
*wR*(*F* ^2^) = 0.112
*S* = 1.14
2108 reflections
172 parameters
H-atom parameters constrained
Δρ_max_ = 0.40 e Å^−3^
Δρ_min_ = −0.34 e Å^−3^

### 

Data collection: *COLLECT* (Hooft, 1998 ▶); cell refinement: *DENZO* (Otwinowski & Minor, 1997 ▶) and *COLLECT*; data reduction: *DENZO* and *COLLECT*; program(s) used to solve structure: *SHELXS97* (Sheldrick, 2008 ▶); program(s) used to refine structure: *SHELXL97* (Sheldrick, 2008 ▶); molecular graphics: *ORTEP-3* (Farrugia, 1997 ▶) and *DIAMOND* (Brandenburg, 2006 ▶); software used to prepare material for publication: *publCIF* (Westrip, 2009 ▶).

## Supplementary Material

Crystal structure: contains datablocks global, I. DOI: 10.1107/S1600536809053343/lh2969sup1.cif

Structure factors: contains datablocks I. DOI: 10.1107/S1600536809053343/lh2969Isup2.hkl

Additional supplementary materials:  crystallographic information; 3D view; checkCIF report

## Figures and Tables

**Table 1:** Hydrogen-bond geometry (Å, °)

*D*—H⋯*A*	*D*—H	H⋯*A*	*D*⋯*A*	*D*—H⋯*A*
N3—H3n⋯O1^i^	0.88	2.26	3.003 (3)	142
N3—H3n⋯N2	0.88	2.41	2.746 (4)	103
C6—H6⋯O1^i^	0.95	2.43	3.214 (4)	140
C10—H10⋯N1^ii^	0.95	2.53	3.448 (4)	162

Symmetry codes: (i) 
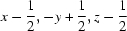
; (ii) 
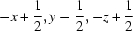
.

**Fig. 1.**
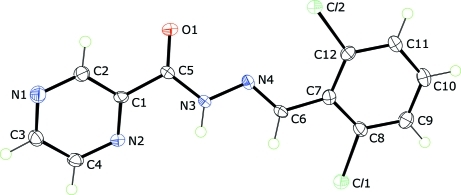
The molecular structure of (I) showing the atom-labelling scheme and displacement ellipsoids at the 50% probability level.

**Fig. 2.**
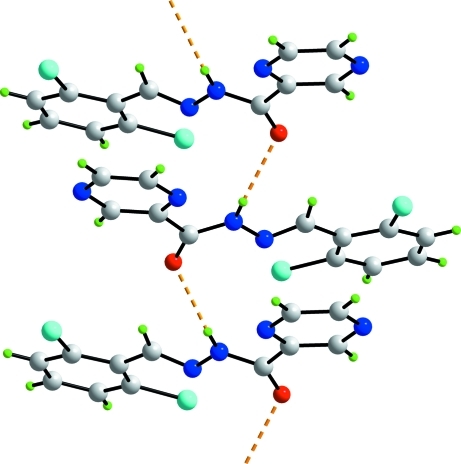
A view of the supramolecular chain in (I) mediated by N–H···O hydrogen bonding (orange dashed lines). Colour code: Cl, cyan; O, red; N, blue; C, grey; and H, green.

**Fig. 3.**
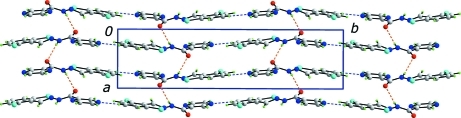
A view of the global crystal packing in (I) with N–H···O hydrogen bonding and C–H···N contacts shown as orange and blue dashed lines, respectively. Colour code: Cl, cyan; O, red; N, blue; C, grey; and H, green.

## supplementary crystallographic information

### Comment

Pyrazine derivatives have various biological activities (Barlin, 1982; Dolezal *et al.*, 2002; Krinkova *et al.*, 2002; Özdemir *et al.*, 2009; Chaisson, *et al.*, 2002; Gordin *et al.*, 2000; de Souza *et al.*,  2005). We have studied the structures of *N*-arylpyrazinecarboxamides (Wardell *et al.*, 2008) and (pyrazinecarbonyl)hydrazones derived from mono-substituted-benzaldehydes (Baddeley *et al.*, 2009). We now report the structure of the title compound, (I).

The molecular structure of (I), Fig. 1, features a planar central C5–N3–N4–C6 core (torsion angle = 176.7 (3)°), but twists are evident in the molecule as evidenced in the O1–C5–C1–N2 and N4–C6–C7–C8 torsion angles of 155.9 (3) and -163.6 (3) °, respectively. This is reflected in the dihedral angle of 12.55 (11) ° formed between the pendant pyrazine and benzene rings. The most prominent intermolecular interactions in the crystal structure involve the amide functionality so that a supramolecular chain mediated by N3–H···O1^i^ [see Table 1 for symmetry codes] interactions is formed, Fig. 2 and Table 1. The chain is stabilized by C6–H···O1^i^ contacts and has base vector [1 0 1]. Interactions of the type C10–H···N1^ii^ are formed orthogonal to the chains formed *via* hydrogen bonding, Table 1. Globally, the molecules pack into layers, in the *ac* plane, and stack along the *b* direction *via* the hydrogen bonding as well π···π interactions [the ring centroid(N1, N2, C1–C4)···ring centroid(C7–C12)^iii^ distance is 3.630 (2) Å with a dihedral angle of 3.28 (17)° for symmetry operation *iii*: 1/2 + *x*, 1/2 - *y*, -1/2 + *z*], Fig. 3.

### Experimental

Solutions of 2-[H_2_NN(*H*)*C*(=*O*)]-pyrazine (0.10 mg, 0.72 mmol) in water (10 ml) and 2,6-dichlorobenzaldehyde (0.125 mg, 0.79 mmol) in ethanol (10 ml) were mixed and the reaction mixture was stirred at ambient temperature until TLC indicated reaction was complete. The solvent was removed under reduced pressure and the residue was washed with cold diethyl ether (30 ml) and recrystallized from ethanol, yield 70%, m.p. 467–469 K. The crystal used in the X-ray structure determination was grown from EtOH solution. ^1^H NMR (400 MHz, DMSO-d_6_) δ: 12.66 (1*H*, s, NH), 9.28 (1*H*, s), 8.95 (1*H*, s, H6), 8.87 (1*H*, s, N=CH), 8.81 (1*H*, s), 7.58 (2*H*, d, J = 8.0 Hz), 7.47 (1*H*, t, J = 8.0 Hz) p.p.m.. ^13^C NMR (100 MHz, DMSO-d_6_) δ: 159.8, 147.9, 145.2, 144.5, 143.3, 134.0, 131.4, 130.6, 129.0 p.p.m.. MS/ESI: [*M* + Na] 317. IR (KBr, cm^-1^) ν: 3240 (N—H); 1675 (C=O).

### Refinement

The N– and C-bound H atoms were geometrically placed (N–H = 0.88 Å and C–H = 0.95 Å) and refined as riding with *U*_iso_(H) = 1.2*U*_eq_(N, C). Owing to a large disparity between *F*_o_ and *F*_c_, the 2 0 0 reflection was omitted in the final cycles of the refinement.

### Crystal data

**Table:**

C_12_H_8_Cl_2_N_4_O	*F*(000) = 600
*M**_r_* = 295.12	*D*_x_ = 1.625 Mg m^−^^3^
Monoclinic, *P*2_1_/*n*	Mo *K*α radiation, λ = 0.71073 Å
Hall symbol: -P 2yn	Cell parameters from 11372 reflections
*a* = 6.9325 (3) Å	θ = 2.9–27.5°
*b* = 24.5997 (13) Å	µ = 0.53 mm^−^^1^
*c* = 7.6136 (4) Å	*T* = 120 K
β = 111.709 (3)°	Plate, colourless
*V* = 1206.31 (10) Å^3^	0.26 × 0.08 × 0.02 mm
*Z* = 4	

### Data collection

**Table:**

Enraf–Nonius KappaCCD area-detector diffractometer	2108 independent reflections
Radiation source: Enraf Nonius FR591 rotating anode	1858 reflections with *I* > 2σ(*I*)
10 cm confocal mirrors	*R*_int_ = 0.052
Detector resolution: 9.091 pixels mm^-1^	θ_max_ = 25.0°, θ_min_ = 3.0°
φ and ω scans	*h* = −8→8
Absorption correction: multi-scan (*SADABS*; Sheldrick, 2007)	*k* = −29→29
*T*_min_ = 0.760, *T*_max_ = 1.000	*l* = −9→8
8211 measured reflections	

### Refinement

**Table:**

Refinement on *F*^2^	Primary atom site location: structure-invariant direct methods
Least-squares matrix: full	Secondary atom site location: difference Fourier map
*R*[*F*^2^ > 2σ(*F*^2^)] = 0.052	Hydrogen site location: inferred from neighbouring sites
*wR*(*F*^2^) = 0.112	H-atom parameters constrained
*S* = 1.14	*w* = 1/[σ^2^(*F*_o_^2^) + (0.0128*P*)^2^ + 2.8931*P*] where *P* = (*F*_o_^2^ + 2*F*_c_^2^)/3
2108 reflections	(Δ/σ)_max_ < 0.001
172 parameters	Δρ_max_ = 0.40 e Å^−^^3^
0 restraints	Δρ_min_ = −0.34 e Å^−^^3^

### Special details

**Table:**

Geometry. All s.u.'s (except the s.u. in the dihedral angle between two l.s. planes) are estimated using the full covariance matrix. The cell s.u.'s are taken into account individually in the estimation of s.u.'s in distances, angles and torsion angles; correlations between s.u.'s in cell parameters are only used when they are defined by crystal symmetry. An approximate (isotropic) treatment of cell s.u.'s is used for estimating s.u.'s involving l.s. planes.
Refinement. Refinement of *F*^2^ against ALL reflections. The weighted *R*-factor *wR* and goodness of fit *S* are based on *F*^2^, conventional *R*-factors *R* are based on *F*, with *F* set to zero for negative *F*^2^. The threshold expression of *F*^2^ > 2σ(*F*^2^) is used only for calculating *R*-factors(gt) *etc*. and is not relevant to the choice of reflections for refinement. *R*-factors based on *F*^2^ are statistically about twice as large as those based on *F*, and *R*- factors based on ALL data will be even larger.

### Fractional atomic coordinates and isotropic or equivalent isotropic displacement parameters (Å^2^)

**Table:**

	*x*	*y*	*z*	*U*_iso_*/*U*_eq_	
Cl1	0.21383 (14)	0.04241 (3)	−0.00147 (12)	0.0280 (2)	
Cl2	0.35539 (13)	0.19347 (3)	0.56395 (11)	0.0216 (2)	
O1	0.4468 (3)	0.31301 (9)	0.2140 (3)	0.0211 (5)	
N1	0.2594 (4)	0.41462 (11)	−0.2597 (4)	0.0229 (6)	
N2	0.2914 (4)	0.30126 (11)	−0.2806 (4)	0.0174 (6)	
N3	0.2987 (4)	0.24065 (10)	0.0247 (4)	0.0169 (6)	
H3N	0.2381	0.2284	−0.0915	0.020*	
N4	0.3326 (4)	0.20678 (11)	0.1769 (4)	0.0177 (6)	
C1	0.3138 (4)	0.32651 (13)	−0.1186 (4)	0.0157 (7)	
C2	0.2997 (5)	0.38272 (13)	−0.1084 (5)	0.0195 (7)	
H2	0.3195	0.3990	0.0102	0.023*	
C3	0.2393 (5)	0.38910 (14)	−0.4200 (5)	0.0216 (7)	
H3	0.2127	0.4101	−0.5312	0.026*	
C4	0.2556 (5)	0.33325 (13)	−0.4310 (4)	0.0202 (7)	
H4	0.2409	0.3172	−0.5488	0.024*	
C5	0.3600 (5)	0.29311 (12)	0.0566 (4)	0.0150 (6)	
C6	0.2611 (5)	0.15869 (13)	0.1347 (4)	0.0171 (7)	
H6	0.1945	0.1492	0.0053	0.020*	
C7	0.2784 (5)	0.11748 (13)	0.2801 (4)	0.0170 (7)	
C8	0.2472 (5)	0.06223 (14)	0.2280 (4)	0.0191 (7)	
C9	0.2446 (5)	0.02134 (14)	0.3508 (5)	0.0231 (7)	
H9	0.2212	−0.0153	0.3084	0.028*	
C10	0.2764 (5)	0.03408 (14)	0.5368 (5)	0.0243 (8)	
H10	0.2743	0.0063	0.6227	0.029*	
C11	0.3110 (5)	0.08741 (13)	0.5960 (5)	0.0198 (7)	
H11	0.3330	0.0963	0.7234	0.024*	
C12	0.3142 (5)	0.12832 (13)	0.4710 (5)	0.0184 (7)	

### Atomic displacement parameters (Å^2^)

**Table:**

	*U*^11^	*U*^22^	*U*^33^	*U*^12^	*U*^13^	*U*^23^
Cl1	0.0433 (5)	0.0230 (5)	0.0214 (5)	−0.0088 (4)	0.0162 (4)	−0.0058 (3)
Cl2	0.0286 (5)	0.0192 (4)	0.0173 (4)	−0.0039 (3)	0.0090 (3)	−0.0028 (3)
O1	0.0247 (12)	0.0195 (12)	0.0159 (12)	−0.0036 (10)	0.0038 (10)	−0.0034 (9)
N1	0.0225 (15)	0.0208 (15)	0.0239 (15)	−0.0005 (12)	0.0067 (12)	0.0021 (12)
N2	0.0185 (14)	0.0170 (14)	0.0149 (13)	−0.0019 (11)	0.0042 (11)	−0.0015 (11)
N3	0.0214 (14)	0.0165 (14)	0.0110 (13)	−0.0020 (11)	0.0036 (11)	0.0011 (10)
N4	0.0210 (14)	0.0170 (14)	0.0150 (14)	0.0006 (11)	0.0066 (12)	0.0035 (11)
C1	0.0103 (15)	0.0203 (17)	0.0128 (16)	−0.0043 (13)	−0.0002 (12)	−0.0008 (12)
C2	0.0185 (16)	0.0174 (16)	0.0199 (17)	0.0018 (13)	0.0038 (14)	0.0000 (13)
C3	0.0198 (17)	0.0225 (18)	0.0193 (17)	−0.0004 (14)	0.0036 (14)	0.0046 (14)
C4	0.0231 (17)	0.0234 (18)	0.0149 (16)	−0.0020 (14)	0.0081 (14)	−0.0027 (13)
C5	0.0150 (15)	0.0174 (16)	0.0120 (15)	0.0017 (13)	0.0044 (13)	0.0008 (12)
C6	0.0174 (16)	0.0203 (17)	0.0106 (15)	0.0001 (13)	0.0018 (13)	0.0020 (13)
C7	0.0133 (15)	0.0187 (16)	0.0188 (16)	0.0006 (13)	0.0056 (13)	0.0026 (13)
C8	0.0196 (17)	0.0232 (17)	0.0161 (17)	−0.0002 (14)	0.0084 (14)	0.0017 (13)
C9	0.0242 (18)	0.0173 (17)	0.0285 (19)	0.0007 (14)	0.0105 (15)	0.0000 (14)
C10	0.0274 (18)	0.0228 (19)	0.0230 (18)	0.0030 (15)	0.0098 (15)	0.0081 (14)
C11	0.0199 (17)	0.0233 (18)	0.0174 (16)	0.0016 (14)	0.0084 (14)	0.0042 (13)
C12	0.0154 (15)	0.0182 (17)	0.0211 (17)	−0.0012 (13)	0.0060 (14)	−0.0013 (13)

### Geometric parameters (Å, °)

**Table:**

Cl1—C8	1.745 (3)	C3—C4	1.384 (5)
Cl2—C12	1.732 (3)	C3—H3	0.9500
O1—C5	1.227 (4)	C4—H4	0.9500
N1—C3	1.333 (4)	C6—C7	1.473 (4)
N1—C2	1.335 (4)	C6—H6	0.9500
N2—C4	1.335 (4)	C7—C12	1.407 (4)
N2—C1	1.338 (4)	C7—C8	1.410 (5)
N3—C5	1.352 (4)	C8—C9	1.378 (5)
N3—N4	1.375 (3)	C9—C10	1.386 (5)
N3—H3N	0.8800	C9—H9	0.9500
N4—C6	1.277 (4)	C10—C11	1.379 (5)
C1—C2	1.390 (4)	C10—H10	0.9500
C1—C5	1.497 (4)	C11—C12	1.391 (4)
C2—H2	0.9500	C11—H11	0.9500
			
C3—N1—C2	115.5 (3)	N4—C6—C7	122.2 (3)
C4—N2—C1	116.0 (3)	N4—C6—H6	118.9
C5—N3—N4	118.8 (3)	C7—C6—H6	118.9
C5—N3—H3N	120.6	C12—C7—C8	115.0 (3)
N4—N3—H3N	120.6	C12—C7—C6	125.5 (3)
C6—N4—N3	114.8 (3)	C8—C7—C6	119.4 (3)
N2—C1—C2	121.8 (3)	C9—C8—C7	123.5 (3)
N2—C1—C5	118.7 (3)	C9—C8—Cl1	116.4 (3)
C2—C1—C5	119.5 (3)	C7—C8—Cl1	120.0 (2)
N1—C2—C1	122.2 (3)	C8—C9—C10	119.4 (3)
N1—C2—H2	118.9	C8—C9—H9	120.3
C1—C2—H2	118.9	C10—C9—H9	120.3
N1—C3—C4	122.7 (3)	C11—C10—C9	119.5 (3)
N1—C3—H3	118.7	C11—C10—H10	120.3
C4—C3—H3	118.7	C9—C10—H10	120.3
N2—C4—C3	121.8 (3)	C10—C11—C12	120.6 (3)
N2—C4—H4	119.1	C10—C11—H11	119.7
C3—C4—H4	119.1	C12—C11—H11	119.7
O1—C5—N3	124.4 (3)	C11—C12—C7	121.9 (3)
O1—C5—C1	121.2 (3)	C11—C12—Cl2	115.6 (2)
N3—C5—C1	114.5 (3)	C7—C12—Cl2	122.5 (2)
			
C5—N3—N4—C6	176.7 (3)	N4—C6—C7—C12	19.9 (5)
C4—N2—C1—C2	0.2 (4)	N4—C6—C7—C8	−163.6 (3)
C4—N2—C1—C5	−178.4 (3)	C12—C7—C8—C9	2.0 (5)
C3—N1—C2—C1	−1.8 (5)	C6—C7—C8—C9	−174.9 (3)
N2—C1—C2—N1	1.3 (5)	C12—C7—C8—Cl1	−176.8 (2)
C5—C1—C2—N1	179.9 (3)	C6—C7—C8—Cl1	6.3 (4)
C2—N1—C3—C4	1.0 (5)	C7—C8—C9—C10	−0.8 (5)
C1—N2—C4—C3	−1.1 (4)	Cl1—C8—C9—C10	178.1 (3)
N1—C3—C4—N2	0.5 (5)	C8—C9—C10—C11	−0.3 (5)
N4—N3—C5—O1	0.7 (5)	C9—C10—C11—C12	0.0 (5)
N4—N3—C5—C1	−179.4 (3)	C10—C11—C12—C7	1.3 (5)
N2—C1—C5—O1	155.9 (3)	C10—C11—C12—Cl2	179.0 (3)
C2—C1—C5—O1	−22.7 (4)	C8—C7—C12—C11	−2.2 (4)
N2—C1—C5—N3	−24.0 (4)	C6—C7—C12—C11	174.4 (3)
C2—C1—C5—N3	157.4 (3)	C8—C7—C12—Cl2	−179.8 (2)
N3—N4—C6—C7	−178.3 (3)	C6—C7—C12—Cl2	−3.1 (5)

### Hydrogen-bond geometry (Å, °)

**Table:**

*D*—H···*A*	*D*—H	H···*A*	*D*···*A*	*D*—H···*A*
N3—H3n···O1^i^	0.88	2.26	3.003 (3)	142
N3—H3n···N2	0.88	2.41	2.746 (4)	103
C6—H6···O1^i^	0.95	2.43	3.214 (4)	140
C10—H10···N1^ii^	0.95	2.53	3.448 (4)	162

Symmetry codes: (i) *x*−1/2, −*y*+1/2, *z*−1/2; (ii) −*x*+1/2, *y*−1/2, −*z*+1/2.
